# Supplementary figures and images for: G protein-coupled receptor kinase-2 (GRK-2) controls exploration through neuropeptide signaling in Caenorhabditis elegans
Source: PLoS Genet. 2023 Jan 18;19(1):e1010613. doi: 10.1371/journal.pgen.1010613 (PMC9886303; doi:10.1371/journal.pgen.1010613)

Figure S1

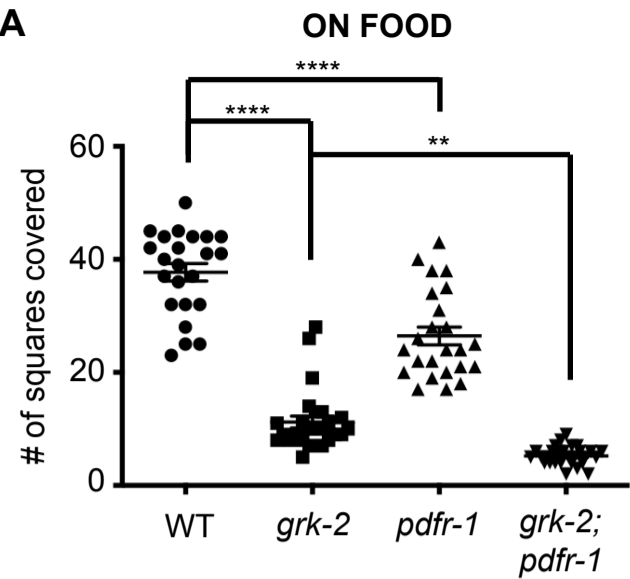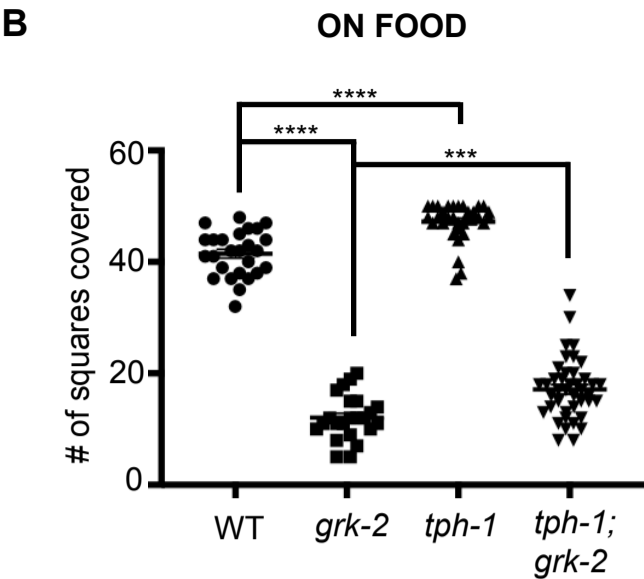

Supplement: S1 Fig — (A) grk-2(gk268) and pdfr-1(ok3425) mutants have exploration defects. The double mutant grk-2(gk268); pdfr-1(ok3425) has a stronger phenotype than the single mutants suggesting that these genes act in parallel to control exploration behavior. Shown is the number of squares (out of 50) single animals covered in ~20 h. (****, P<0.0001. **, P<0.01. One way ANOVA followed by a Bonferroni test. Error bars = SEM; n = 23–28). (B) grk-2(gk268) mutant animals explore less and tph-1(mg280) more than WT animals. The double mutant tph-1(mg280); grk-2(gk268) has an intermediate phenotype suggesting that these genes act in parallel pathways to control exploration behavior. (****, P<0.0001. ***, P<0.001. One way ANOVA followed by a Bonferroni test. Error bars = SEM; n = 23–41). (PDF) [file pgen.1010613.s001.pdf]

Figure S2

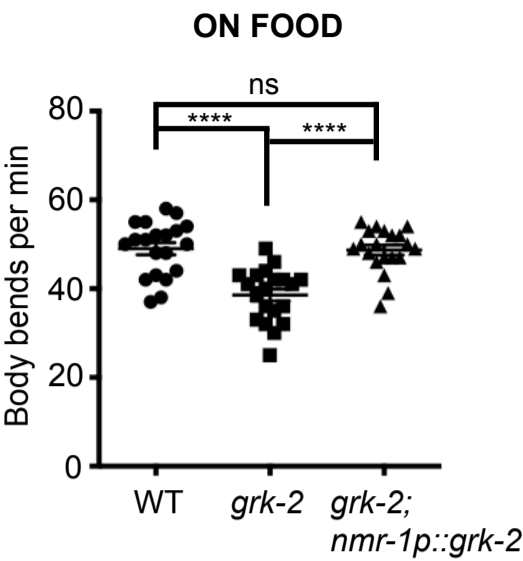

Supplement: S2 Fig — grk-2 cDNA expression in command interneurons (Pnmr-1, transgene yakSi32) is sufficient to rescue the slow locomotion of grk-2(gk268) mutant animals. (****, P<0.0001. ns, P>0.05. One way ANOVA followed by a Bonferroni test. Error bars = SEM; n = 20). (PDF) [file pgen.1010613.s002.pdf]

Figure S3

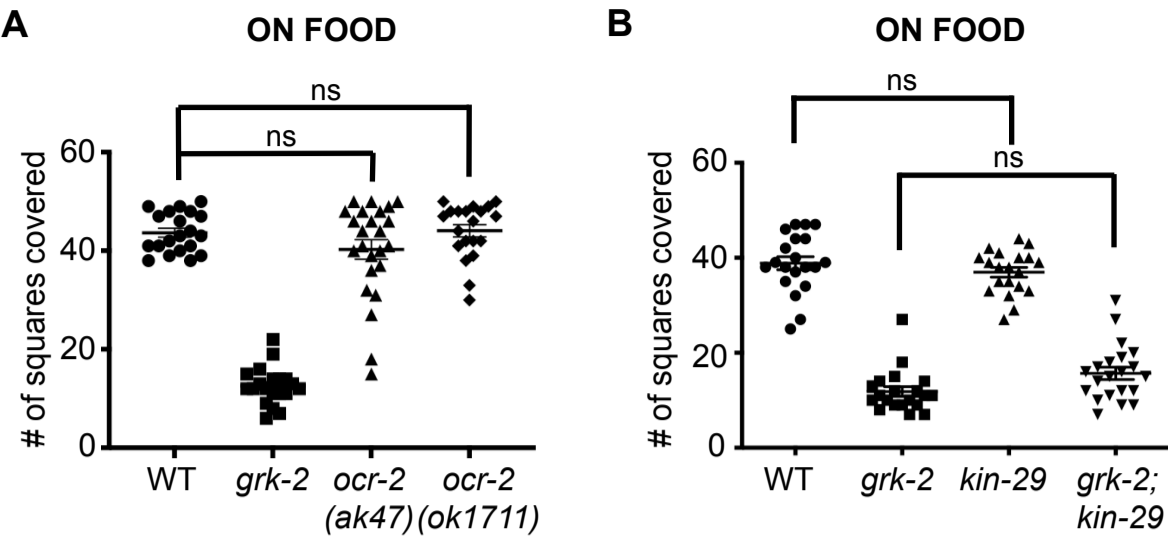

Supplement: S3 Fig — (A) ocr-2(ak47), ocr-2(ok1717) (strains BJH2763 and BJH2762, respectively) and kin-29(oy38) mutants do not have exploration defects. Shown is the number of squares (out of 50) single animals covered in ~20 h. (ns, P>0.05. One way ANOVA followed by a Bonferroni test. Error bars = SEM; n = 20–24). (B) kin-29(oy38) mutant animals do not have an exploration defect and the double mutant grk-2(gk268); kin-29(oy38) has an exploration defect similar to grk-2(gk268). (ns, P>0.05. One way ANOVA followed by a Bonferroni test. Error bars = SEM; n = 20–21). (PDF) [file pgen.1010613.s003.pdf]

Figure S4

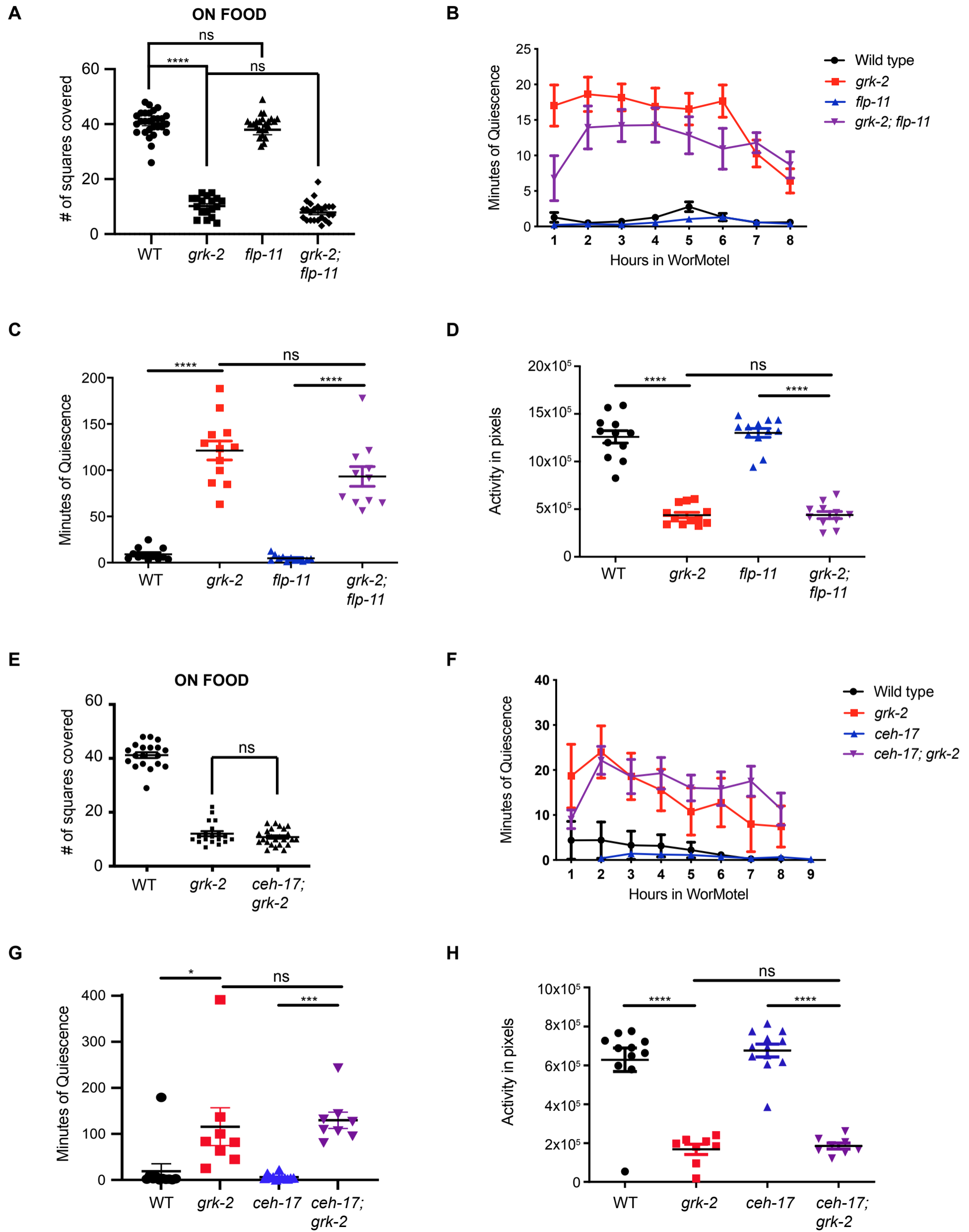

Supplement: S4 Fig — (A) The flp-11(tm2706) mutation does not affect the exploration defect of grk-2(gk268) mutant animals. Shown is the number of squares (out of 50) single animals covered in ~20 h. (****, P<0.0001. ns, P>0.05. One way ANOVA followed by a Bonferroni test. Error bars = SEM; n = 20–28). The data points shown for WT and grk-2 mutant are the same as in Fig 6D (these experiments were run in parallel). (B) grk-2(gk268) mutant animals show enhanced movement quiescence and the flp-11(tm2706) mutation does not affect this phenotype. The graph shows the average minutes animals spent in quiescence per hour in the WorMotel. Quiescence is the time when there were zero pixels moved between two frames separated by 10 sec (See Methods). (C) The graph shows the total minutes individual animals spent in quiescence in 8h in the WorMotel. (****, P<0.0001. ns, P>0.05. One way ANOVA. Error bars = SEM; n = 11–12). (D) grk-2(gk268) mutant animals show reduced activity and the flp-11(tm2706) mutation does not affect this phenotype. The graph shows the activity in pixels of individual animals in 8 h in the WorMotel. Activity is the number of pixels moved between frames separated by 10 sec. (****, P<0.0001. ns, P>0.05. One way ANOVA. Error bars = SEM; n = 11–12). (E) The ceh-17(np1) mutation does not affect the exploration defect of grk-2(gk268) mutant animals. Shown is the number of squares (out of 50) single animals covered in ~20 h. (ns, P>0.05. One way ANOVA. Error bars = SEM; n = 20–24). (F) grk-2(gk268) mutant animals show enhanced movement quiescence, and the ceh-17(np1) mutation does not affect this phenotype. (G) The graph shows the total minutes individual animals spent in quiescence in 8 h in the WorMotel. (*, P<0.05. ***, P<0.001. ns, P>0.05. One way ANOVA. Error bars = SEM; n = 8–12). (H) The graph shows the activity in pixels of individual animals in 8 h in the WorMotel. (****, P<0.0001. ns, P>0.05. Error bars = SEM; n = 8–12). (PDF) [file pgen.1010613.s004.pdf]

Figure S5

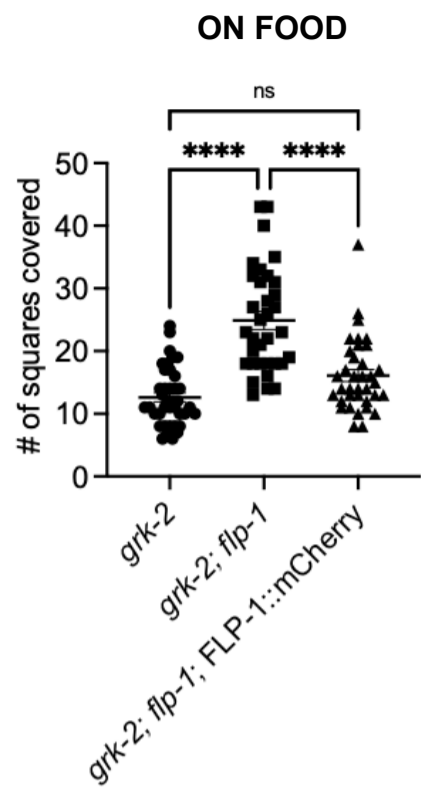

Supplement: S5 Fig — Shown is the number of squares (out of 50) single animals covered in ~20 h. (****, P<0.0001. ns, P>0.05. One way ANOVA followed by a Bonferroni test. Error bars = SEM; n = 34–39). (PDF) [file pgen.1010613.s005.pdf]
