# Supplementary material for: G protein-coupled receptor kinase-2 (GRK-2) controls exploration through neuropeptide signaling in Caenorhabditis elegans
Source: PLoS Genet. 2023 Jan 18;19(1):e1010613. doi: 10.1371/journal.pgen.1010613 (PMC9886303; doi:10.1371/journal.pgen.1010613)
Supplement: S2 Table — (DOCX) [file pgen.1010613.s007.docx]

**S2 Table. List of plasmids**

Gateway destination vectors

pCFJ150 Gateway destination vector for insertion at chr II Mos site *ttTi5605*

Gateway entry clones

BJP-T11 *dat-1p* [4-1] (690 bp of the *dat-1* promoter upstream of the ATG)

BJP-C664 *twk-47p* [4-1] (222 bp of the *twk-47* promoter upstream of the ATG)

pADA126 *let-858* 3’UTR [2-3]

pCFJ31 *acr-2p* [4-1] (3362 bp of the *acr-2* promoter upstream of the ATG)

pCFJ326 *tbb-2* 3’UTR::OPERON::GFP [2-3]

pCFJ1973 mNEON-NLS [1-2]

pCR185 GFP::*unc-54* 3’UTR [2-3]

pEGB05 *rab-3p* [4-1] (1224 bp of the *rab-3* promoter upstream of the ATG)

pET68 *grk-2* cDNA [1-2]

pET85 *osm-6p* [4-1] (2400 bp of the *nmr-1* promoter upstream of the ATG)

pET89 *grk-2p* [4-1] (2895 bp of the *grk-2* promoter upstream of the ATG)

pET108 *xbx-1p* [4-1] (425 bp of the *xbx-1* promoter upstream of the ATG)

pET276 *odr-3p* [4-1] (4125 bp of the *odr-3* promoter upstream of the ATG)

pET299  *gcy-8p* [4-1] (1923 bp of the *gcy-8* promoter upstream of the ATG)

pET312 *flp-1* cDNA [1-2]

pET332 *flp-1p* [4-1] (514 bp of the *flp-1* promoter upstream of the ATG)

pET336 *flp-1* ORF [1-2]

pGH107 tagRFP::*let-858* 3’UTR [2-3]

pIR47 *str-1p* [4-1] (4012 bp of the *str-1* promoter upstream of the ATG)

pIR211  *str-2p* [4-1] (2000 bp of the *str-2* promoter upstream of the ATG)

pIR419  *odr-10p* [4-1] (1000 bp of the *odr-10* promoter upstream of the ATG)

pJB-GL24 *sra-6p* [4-1] (2963 bp of the *sra-6* promoter upstream of the ATG)

pMA102 *nmr-1p* [4-1] (4709 bp of the *nmr-1* promoter upstream of the ATG)

Gateway expression constructs

pET79 *rab-3p::grk-2 cDNA:tbb-2* 3’UTR::OPERON::GFP_pCFJ150

pET83 *acr-2p::grk-2 cDNA::tbb-2* 3’UTR::OPERON::GFP_pCFJ150

pET86 *osm-6p::grk-2 cDNA::tbb-2* 3’UTR::OPERON::GFP_pCFJ150

pET90 *grk-2p::grk-2 cDNA*::GFP_pCFJ150

pET91 *grk-2p::grk-2 cDNA*::tagRFP_pCFJ150

pET109 *xbx-1p::grk-2 cDNA::tbb-2* 3’UTR::OPERON::GFP_pCFJ150

pET119 *nmr-1p::grk-2 cDNA::tbb-2* 3’UTR::OPERON::GFP_pCFJ150

pET278 *odr-3p::grk-2 cDNA::tbb-2* 3’UTR::OPERON::GFP_pCFJ150

pET285 *sra-6p::grk-2 cDNA::tbb-2* 3’UTR::OPERON::GFP_pCFJ150

pET286 *odr-10p::grk-2 cDNA::tbb-2* 3’UTR::OPERON::GFP_pCFJ150

pET289 *str-1p::grk-2 cDNA::tbb-2* 3’UTR::OPERON::GFP_pCFJ150

pET291 *str-2p::grk-2 cDNA::tbb-2* 3’UTR::OPERON::GFP_pCFJ150

pET304 *gcy-8p::grk-2 cDNA::tbb-2* 3’UTR::OPERON::GFP_pCFJ150

pET307 *odr-3p::mNeon::NLS::let-858* 3’UTR_pCFJ150

pET308 *sra-6p::npr-1 cDNA::let-858* 3’UTR_pCFJ150

pET320 *twk-47p*::*flp-1* cDNA::*tbb-2* 3’UTR::OPERON::GFP_pCFJ150

pET326 *osm-6p::egl-4 cDNA::tbb-2* 3’UTR::OPERON::GFP_pCFJ150

pET344 *flp-1p*::*flp-1* cDNA::*tbb-2* 3’UTR::OPERON::GFP_pCFJ150

pET346 *flp-1p*::FLP-1 ORF::GFP_pCFJ150

pET362 *hsp-16.41p::grk-2* cDNA::*tbb-2* 3’UTR::OPERON::GFP_pCFJ150

pET370 *dat-1p*::*grk-2* cDNA::*tbb-2* 3’UTR::OPERON::GFP_pCFJ150

Gifts

pEM01  *flp-21p::loxP::STOP::loxP::npr-1 cDNA::SL2::GFP* (a gift from Cori Bargmann)

pEM03 *ncs-1p*::nCre (a gift from Cori Bargmann)

pCS232 *flp-1(trc)p::*FLP-1::mCherry (a gift from Alexander Gottschalk)
