# Supplementary material for: G protein-coupled receptor kinase-2 (GRK-2) controls exploration through neuropeptide signaling in Caenorhabditis elegans
Source: PLoS Genet. 2023 Jan 18;19(1):e1010613. doi: 10.1371/journal.pgen.1010613 (PMC9886303; doi:10.1371/journal.pgen.1010613)
Supplement: Attachment — Submitted filename: Response to Reviewers.pdf [file pgen.1010613.s009.pdf]

**Point by point response: "G Protein-Coupled Receptor Kinase-2 (GRK-2) regulates exploration through neuropeptide signaling in *Caenorhabditis elegans*"**

**Editor's comments:**

Dear Dr. Topalidou,

Thank you very much for submitting your Research Article entitled 'G Protein-Coupled Receptor Kinase-2 (GRK-2) regulates exploration through neuropeptide signaling in Caenorhabditis elegans' to PLOS Genetics.

The manuscript was fully evaluated at the editorial level and by independent peer reviewers. The reviewers appreciated the attention to an important topic but identified some concerns that we ask you address in a revised manuscript.

We therefore ask you to modify the manuscript according to the review recommendations. Your revisions should address the specific points made by each reviewer. Please pay particular attention to the reviewer points about a potential molecular model. For example, two reviewers raised questions about the grk-2 and egl-4 interaction. Improvements to Figure 9 could also be helpful.

Dear Editor,

We modified the manuscript according to the recommendations made by the reviewers. Below we include a point-by-point response to each individual comment. As suggested by the editor, we made sure to change the discussion, as well as the model figure (new Fig 9), by including a possible molecular model on (1) how GRK-2 and EGL-4 might act in opposite ways to regulate exploration and (2) how GRK-2 might act in sensory neurons to regulate FLP-1 secretion from AVK interneurons.

Specifically, to address the reviewer's points about a potential molecular model we:

1. Changed Fig 9 (see new Fig. 9) as well as the figure legend.
2. Changed the discussion by adding the following points:

Line 434: "Given that expression of EGL-4 in the ciliated neurons reverses this suppression, we propose that both GRK-2 and EGL-4 act in ciliated neurons to control exploration but in opposite ways. We do not yet know whether the two kinases act in the same ciliated neurons to control exploration. Given that both GRK-2 and EGL-4 act in multiple ciliated neurons to regulate exploration and that other chemosensory and osmosensory mutants have similar exploration defects as *grk-2*, we support a model in which GRK-2 acts in ciliated neurons to positively control sensory perception and affect the exploration state of the animal by regulating EGL-4. We propose that in *grk-2* and cilium structure mutants, impaired sensory perception leads to increase in EGL-4 activity and decreased exploration. A similar model was supported by previous studies, which showed that EGL-4 regulates body size and locomotory states in a way depending on sensory perception [2]. Alternatively, the two kinases GRK-2 and EGL-4, might act in different ciliated neurons, leading to opposite outcomes in sensory perception and exploration."

Line 447: "In previous work we have shown that GRK-2 regulates crawling and swimming [27,28] by possibly phosphorylating the D2-like dopamine receptor DOP-3 in premotor

interneurons (Fig 9A). In the case of chemosensation and exploration, the target(s) of GRK-2, which are located in chemosensory neurons and not interneurons, are currently unknown (Fig 9B). Similarly, we do not yet know the identity of the phosphorylation target(s) for EGL-4."

Line 462: "This result suggests that GRK-2 and EGL-4 act antagonistically to control movement quiescence outside lethargus. We propose that GRK-2 acts in ciliated neurons to control sensory perception and affect movement quiescence by negatively regulating EGL-4."

Line 507: "Since GRK-2 acts in ciliated neurons to affect exploration and GRK-2 modulates FLP-1 secretion from AVK neurons we propose that GRK-2 acts in ciliated neurons to mediate FLP-1 secretion from AVKs in a non-cell autonomous way. Given that there is no known synaptic input from chemosensory neurons to AVKs [67] we suggest that GRK-2-driven regulation of sensory neuron activity affects a neuromodulatory pathway that drives FLP-1 secretion from AVKs (Fig 9B). Alternatively, GRK-2 action in ciliated neurons may lead to modulation of the neuronal circuit downstream of the ciliated neurons that eventually affects secretion of FLP-1. According to our model, GRK-2 activity positively regulates chemosensory neuron activity, which in turn inhibits EGL-4 activity, modulating exploration and the arousal state of *C. elegans* through neuromodulatory signaling acting in interneurons (Fig 9B)."

Line 1031: "GRK-2 positively regulates (A) crawling and swimming by acting in premotor interneurons in a manner that depends on the D2-like dopamine receptor DOP-3 [27,28] and (B) exploration by acting in ciliated neurons in an opposite way from EGL-4. We propose a model in which GRK-2 acts in multiple ciliated sensory neurons to positively control sensory perception and (1) inhibit the activity of the EGL-4 kinase in ciliated neurons, (2) negatively regulate the neuropeptide receptor NPR-1 in RMG interneurons, and (3) negatively control the levels of FLP-1 secretion from AVK interneurons. Since the ciliated neurons and AVK interneurons do not have direct connections, we propose that GRK-2 controls FLP-1 secretion from AVK interneurons in a non-cell autonomous way through a neuromodulatory pathway. Proteins that positively regulate crawling, swimming, or exploration are shown in green and proteins that negatively regulate crawling, swimming, or exploration are shown in red. Dashed lines show indirect interactions."

**Reviewer's comments:**

**Reviewer #1**: In this manuscript, Davis et al find that GRK-2, G protein-coupled receptor kinase-2, regulates exploration behavior in the nematode C. elegans. Using genetics, several nicely quantified behavioral assays, cell-specific rescue and imaging, they show that GRK-2 functions in several sensory neurons, opposite of the cGMP-dependent protein kinase EGL-4, the neuropeptide receptor NPR-1 and the neuropeptides FLP-1 and FLP-18. Their work is very important for understanding C. elegans behavior, where many chemotaxis or learning and memory behavioral assays use a readout based on locomotion and are thus affected by the arousal state of the animal. In addition, as all the proteins involved are strongly conserved, similar pathways might be involved in the regulation of locomotion behavior in other animals. The manuscript is well written, data has been clearly presented and experimental methods, including statistical analysis, has been clearly described.

We thank the reviewer for the positive comments on our manuscript.

Here are my comments: Page 7-8, lines 139-154. In this paragraph the authors discuss the results that suggest that GRK-2 acts in a different pathway than serotonin and PDF signaling. However, in the final sentence of the paragraph, the authors conclude that GRK-2 acts in a

different pathway. I think this conclusion is too strong and should be rephrased to something like "Our results suggest that GRK-2 acts in a different pathway…". The same applies to the last paragraph of the results, page 16. Also, there the conclusion is too strong and should be rephrased.

We changed the text according to the reviewer's instructions.

Line 154: "Our results imply that GRK-2 acts in a different pathway…"

Line 392: "These results altogether indicate…"

On page 9, the authors discuss the cell-specific rescue experiments to find out in which sensory neurons GRK-2 functions to regulate exploration. They show data where *grk-2* was expressed in AWA, AWB and AWC-ON, using three different promoters, but they don't show to what extent each of these promoters rescue the phenotype. I would strongly urge the authors to do these experiments and report on them, because it could reveal whether expression in either of these cells, or only in one particular cell is sufficient for rescue.

We injected the individual rescuing constructs in *grk-2(gk268)* mutant animals and observed that *grk-2* expression by each individual promoter only slightly rescued the *grk-2* exploration defect, suggesting that *grk-2* acts in more than a single set of ciliated neurons to control exploration. The results of these experiments are now included in new Fig 2F.

Line 191: "Similarly, *grk-2* expression in the AWA, AWB, or AWC neurons (*odr-10p*: expression in AWA [41]; *str-1p*: expression in AWB [42]; *str-2p*: expression in AWC-ON [43]) only slightly rescued the *grk-2* exploration defect (Fig 2F)."

The rescue experiments often show only partial rescue. This could be because the construct was not expressed in all cells required for full rescue, but it could also be that the level of expression was either too low or too high. Are there reasons to believe that *grk-2* overexpression could cause a defect? Please discuss this possibility.

We agree with the reviewer that partial rescue could indicate issues with the expression, such as too low or too high expression or partial expression. The fact that transgenic expression of *grk-2* under *rab-3, xbx-1*, or *osm-6* heterologous promoters fully rescued the *grk-2* exploration defect (Fig 2A-C) argues against the possibility that the precise level of GRK-2 is critical to its function in exploration. To verify that the partial rescue observed was not the result of lack of or partial expression an operon GFP was included in the *grk-2* expression constructs downstream of the 3'UTR allowing confirmation of expression (see Methods).

We changed the text in order to discuss these possibilities.

Line 172: "Our result that transgenic expression of *grk-2* under *rab-3, xbx-1*, or *osm-6* heterologous promoters fully rescued the *grk-2* exploration defect (Fig 2A-C) suggests that the precise level of GRK-2 is not critical to its function."

Line 179: "An operon GFP was included in the expression construct downstream of the 3'UTR allowing confirmation of expression (see Methods)."

Line 530: "For the cell-specific rescuing experiments, an operon GFP was included in the expression constructs downstream of the 3'UTR [70]. This resulted in expression of untagged

protein, but also allowed for confirmation of proper promoter expression by monitoring GFP expression."

On page 10 the authors show data that suggest that *che-2* acts in the same pathway as *grk-2*, as double mutants have the same phenotype as the two singles. It would be good to strengthen this finding, as the authors show the results of only one double and one assay. It would be good to also test the *che-2 grk-2* double mutants in the off-food assay, and if possible, it would be nice to test whether the same holds true for another cilium mutant.

We performed the experiments that the reviewer suggested and included them in new FIG 3B,C. First, we tested the exploration behavior of a different cilium structure mutant (*daf-10(e1387)*) and found that the phenotype of the double mutant *grk-2; daf-10* is similar to that of the single mutants *grk-2* or *daf-10*. Second, we tested *daf-10* and *che-2* as well as the double mutants *grk-2; che-2 and grk-2; daf-10* in the off-food dispersal assay and found that the single mutants (*grk-2, che-2*, or *daf-10*) behave similarly to the double mutants (*grk-2; che-2* or *grk-2; daf-10*). Both these experiments support our model that the cilium structure mutants *daf-10* and *che-2* act in the same pathway as *grk-2* to regulate exploration.

Line 215: "To confirm that cilium structure mutants have exploration defects similar to *grk-2*, we performed the exploration assay on *che-2(e1033)* and *daf-10(e1387)* mutant animals in the presence or absence of food. We found that *che-2(e1033)* and *daf-10(e1387)* mutant animals are exploration defective identical to *grk-2(gk268*) (Fig 3A-C). Additionally, the *che-2(e1033)* and *daf-10(e1387)* mutations do not enhance the phenotype of *grk-2* mutant (Fig 3A-C). The lack of an obvious enhancement is not due to a floor effect since *grk-2; pdfr-1* double mutant animals have stronger exploration phenotype than the single mutants (Fig S1A). These findings suggest that *che-2, daf-10*, and *grk-2* act in the same pathway to regulate exploration (Fig 3A-C)."

On page 11, the authors state that EGL-4 promotes dwelling as loss of function mutants explore more than wt animals. However, I don't see this effect in the assays that the authors show. Please explain.

We agree with the reviewer that in the experiment shown in new Fig 4A, *egl-4* mutant animals do not seem to explore significantly more than wild type animals. To examine whether this could be due to a ceiling effect we repeated the assay in larger plates (10 cm). As shown in new Fig 4B, using 10 cm plates we confirmed that *egl-4* mutant animals explore more than wild type animals. We describe this experiment in the "Results" section:

Line 256: "We noticed that when the exploration assay was performed on 6 cm plates, *egl-4* mutant animals didn't seem to explore significantly more than wild type animals, probably due to a ceiling effect (Fig 4A). In contrast, when the assay was performed on 10 cm plates, *egl-4* mutant animals explored significantly more than wild type animals (Fig 4B)."

Line 919: "(A), (B) The *egl-4(n479)* mutation partially suppresses the exploration defect of *grk-2(gk268)* mutant as assayed on 6 cm (A) and 10 cm (B) plates. Shown is the number of squares (out of 50, (A) or 150, (B)) single animals covered in ~20 h. Please note that *egl-4* mutant animals explore more than wild type animals as assayed on 10 cm plates."

Line 253 refers to fig 4D. Should this be fig 4B-D?

We thank the reviewer for this correction.

Line 272: (Fig 4D-F)

Fig 6A and B shows that *npr-1* mutants explore more than wt. Fig 6A shows the result of assays on 6 cm plates, whereas 6B shows results of assays done on 10 cm plates. This is not discussed in the text. Please explain why this is done. And it is unclear to me why the 10 cm assays have not been done for other mutants.

The reason why we performed the exploration assay using 10 cm plates is to avoid a ceiling effect in the case of mutants that explore more than wild type (e.g. *npr-1, egl-4*). We explain this in the revised manuscript. We also included data showing the exploration behavior of *egl-4* mutant animals on 10 cm plates (Fig. 4B). We didn't consider important to perform the exploration assay on 10 cm plates for mutants that do not explore more than WT.

Line 256: "We noticed that when the exploration assay was performed on 6 cm plates, *egl-4* mutant animals didn't seem to explore significantly more than wild type animals, probably due to a ceiling effect (Fig 4A). In contrast, when the assay was performed on 10 cm plates, *egl-4* mutant animals explored significantly more than wild type animals (Fig 4B)."

Line 542: "The exploration assay is an established assay that measures the ability of the animals to explore (wander) during long periods of time [4]. Exploration assays were performed on 6 or 10 cm nematode growth medium (NGM) plates uniformly seeded with 200 or 500 ul of the OP50 strain of *E. coli*, respectively, and spread with a glass spreader. 10 cm plates were used in the case of mutants that explore more than wild type animals (e.g. *egl-4, npr-1*) in order to avoid a ceiling effect because wild type animals usually explore most of the area of a 6 cm plate in a 20h period."

Line 919: "(A), (B) The *egl-4(n479)* mutation partially suppresses the exploration defect of *grk-2(gk268)* mutant as assayed on 6 cm (A) and 10 cm (B) plates. Shown is the number of squares (out of 50, (A) or 150, (B)) single animals covered in ~20 h. Please note that *egl-4* mutant animals explore more than wild type animals as assayed on 10 cm plates."
Line 964: "The *npr-1(ky13)* mutation suppresses the exploration defect of *grk-2(gk268)* mutant as assayed on 10 cm plates."

Fig. 6E shows that mutation of *flp-21* does not suppress the effect of *grk-2* loss of function and the authors conclude that flp-21 does not play a role in this phenotype. However, we see quite often that phenotypes are masked by functional redundancy. I was wondering whether the authors considered testing *grk-2 flp-18 flp-21* triple mutants. These mutants might reveal a function of *flp-21*.

To examine whether *flp-18* and *flp-21* have a redundant function in GRK-2-mediated exploration we created the triple mutant *grk-2; flp-21; flp18* and tested its exploration phenotype. We found that *flp-21; flp-18* mutants, were not stronger suppressors of the *grk-2* exploration defect than *flp-18* mutants, suggesting that the *flp-21* mutation does not act redundantly with *flp-18*. These data are included in new Fig 6E.

Line 321: "The *flp-21* mutation did not affect the *grk-2* exploration defect and *flp-21; flp-18* mutants were not stronger suppressors than *flp-18* mutants, suggesting that the *flp-21(ok889)* mutation does not suppress the *grk-2* exploration defect (Fig 6E)."

Fig 8c shows the results of a control experiment, which is not mentioned or explained in the text. Please explain this experiment briefly."

We followed the reviewer's suggestion and explained the control experiment in the text.

Line 383: "To examine whether background fluorescence in the coelomocytes of *grk-2; flp-1* mutants also changed, together with FLP-1::mCherry we co-expressed a reporter expressing GFP driven by the coelomocyte-specific promoter *unc-122* and tested the levels of GFP fluorescence in the coelomocytes of *flp-1* and *grk-2; flp-1* mutants. We found that GFP fluorescence remained unchanged in *grk-2; flp-1* mutants suggesting that the increase in FLP-1::mCherry fluorescence in *grk-2; flp-1* coelomocytes is specific to FLP-1::mCherry (Fig 8C)."

**Reviewer #2**: This paper is a nice genetic dissection of multiple intersecting pathways that regulate locomotion and sleep in *C. elegans*.

We thank the reviewer for the positive comments on our article.

There are many parameters regulating these behaviors: three, roaming/exploration, dwelling, and quiescence, were examined. Previous work has shown that the cGMP-dependent protein kinase EGL-4 regulates the time in quiescence, roaming, and dwelling. For instance, activation of EGL-4 in sensory neurons regulates dwelling.

The authors' previous work showed that the GRK-2 kinase in premotor interneurons promotes crawling and swimming by antagonizing dopamine DOP-3 receptor signaling. In this paper, the authors confirmed previous work that loss of grk-2 resulted in decreased exploration in the presence or absence of food. However, the decreased exploration was independent of DOP-3 receptor signaling, suggesting that a pathway independent of dopamine signaling is activated during exploration. Using cell-specific promoters to drive grk-2, the authors determined that grk-2 function in specific ciliated sensory neurons (AWA, AWB, AWC, ASH, ADF) partially regulates exploration and acts antagonistically to EGL-4 signaling in the same sensory neurons.

Through epistasis analysis, the authors eliminated (e.g., serotonin or PDF signaling) or identified several pathways in which GRK-2 acts. The decreased exploration of grk-2 mutants is linked to increased sleep, which is partially dependent on the activity of the APTF-1 transcription factor in an unknown cell; loss of aptf-1 partially suppressed the exploration defect in grk-2 mutants but some unknown mechanism. Similarly, loss of FLP-18 release, through unknown cells (sensory neurons, interneurons?) results in loss of signaling through the NPR-1 receptor in RMG neurons, which partially suppressed the exploration defect in grk-2 mutants.

Loss of *flp-1* partially suppressed the exploration defect of *grk-2* mutants. Expression of flp-1 in the AVK interneurons rescued these defects in the *grk-2; flp-1* mutants, suggesting that release of FLP-1 peptides affects the exploration behavior. In epistasis analysis, FLP-1 acts in the same pathway as NPR-1; the likely FLP-1 receptor is FRPR-7, which is expressed in many neurons and the coelomocytes. Secretion of FLP-1 peptides was monitored by mCherry presence in coelomocytes; however, FRPR-7 is also expressed in coelomocytes, which slightly muddles the uptake experiments. In addition, the mCherry appeared to be in-frame with the last FLP-1 gene peptide PNFLRFG, which would inactivate this peptide because of the large size of the mCherry relative to the peptide. Furthermore, it was unclear whether this transgene rescued the exploration defect of *grk-2* mutants and/or whether the mCherry tag affected the way the FLP-1

propeptide is cleaved. These questions should be addressed either in the Results, Discussion, or both.

The FLP-1::mCherry secretion assay that we used to measure FLP-1 secretion from AVK neurons was previously used by Oranth et al, (2018) as a way of evaluating FLP-1 secretion. Oranth et al. (2018) showed that FLP-1::mCherry was found in AVK cell bodies and processes, as well as in coelomocytes, and that mCherry secretion followed dense core vesicle release. Oranth et al. (2018) did not address whether FLP-1::mCherry is a rescuing construct. To address this and the reviewer's point, we tested whether FLP-1::mCherry could reverse the *flp-1* suppression of the exploration phenotype of *grk-2* mutants. As shown in new Fig S5C, FLP-1::mCherry reversed the *flp-1* suppression of the exploration defect of *grk-2*. We have included these new data in our revised manuscript.

Line 377: "Previous work confirmed that FLP-1::mCherry follows the dense core vesicle route for neuropeptide secretion and used the FLP-1::mCherry secretion assay as a way of evaluating FLP-1 secretion [36]. Notably, FLP-1::mCherry expression in *grk-2; flp-1* reversed the suppression of the exploration defect of *grk-2* by *flp-1,* suggesting that FLP-1 in the FLP-1::mCherry reporter retains its function in exploration (Fig S5)."

We think that the site of action of FRPR-7 to explore exploration is out of the scope of this paper and we hope that this will be included in a follow up study. For this reason, we decided not to focus on the expression pattern and site of action of FRPR-7 in this manuscript.

The Discussion should be better integrated with a molecular model. The first few pages discussed epistatic relationships without mentioning the proteins, whose molecular identities are known. GRK-2, like EGL-4, is a kinase. Its function is to phosphorylate, but the question is what its target is within the same cells as to where EGL-4 is expressed. Presumably, the two kinases are phosphorylating different targets, thereby activating different pathways leading to different exploration results; however, this was not clearly indicated, particularly in Fig. 9, which was confusing. Are the authors proposing that grk-2 and egl-4 are expressed in the same sensory neurons or different sensory neurons? The arrows and inhibitory arrows are pointing to how proteins are interacting. For example, how does GRK-2, a kinase that is expressed in sensory neurons, negatively regulate FLP-1, which is expressed in the AVK interneuron? If the site of action of the gene is known, it should be so represented in Fig. 9.

Overall, this paper assigned different protein activities to different pathways that modulate different behaviors. The genetics was very well done and highlighted interesting aspects of behavior. The paper suffers from not integrating the work better, particularly in the Discussion.

We thank the reviewer for prompting us to alter both the discussion and the model (new Fig 9) by including a mechanistic explanation and a possible model on (1) how GRK-2 and EGL-4 might act in opposite ways to regulate exploration and (2) how GRK-2 might act in sensory neurons to regulate FLP-1 secretion from AVK interneurons.

Line 434: "Given that expression of EGL-4 in the ciliated neurons reverses this suppression, we propose that both GRK-2 and EGL-4 act in ciliated neurons to control exploration but in opposite ways. We do not yet know whether the two kinases act in the same ciliated neurons to control exploration. Given that both GRK-2 and EGL-4 act in multiple ciliated neurons to regulate exploration and that other chemosensory and osmosensory mutants have similar exploration defects as *grk-2*, we support a model in which GRK-2 acts in ciliated neurons to positively control sensory perception and affect the exploration state of the animal by regulating EGL-4.

We propose that in *grk-2* and cilium structure mutants, impaired sensory perception leads to increase in EGL-4 activity and decreased exploration. A similar model was supported by previous studies, which showed that EGL-4 regulates body size and locomotory states in a way depending on sensory perception [2]. Alternatively, the two kinases GRK-2 and EGL-4, might act in different ciliated neurons, leading to opposite outcomes in sensory perception and exploration."

Line 447: "In previous work we have shown that GRK-2 regulates crawling and swimming [27,28] by possibly phosphorylating the D2-like dopamine receptor DOP-3 in premotor interneurons (Fig 9A). In the case of chemosensation and exploration, the target(s) of GRK-2, which are located in chemosensory neurons and not interneurons, are currently unknown (Fig 9B). Similarly, we do not yet know the identity of the phosphorylation target(s) for EGL-4."

Line 462: "This result suggests that GRK-2 and EGL-4 act antagonistically to control movement quiescence outside lethargus. We propose that GRK-2 acts in ciliated neurons to control sensory perception and affect movement quiescence by negatively regulating EGL-4."

Line 507: "Since GRK-2 acts in ciliated neurons to affect exploration and GRK-2 modulates FLP-1 secretion from AVK neurons we propose that GRK-2 acts in ciliated neurons to mediate FLP-1 secretion from AVKs in a non-cell autonomous way. Given that there is no known synaptic input from chemosensory neurons to AVKs [67] we suggest that GRK-2-driven regulation of sensory neuron activity affects a neuromodulatory pathway that drives FLP-1 secretion from AVKs (Fig 9B). Alternatively, GRK-2 action in ciliated neurons may lead to modulation of the neuronal circuit downstream of the ciliated neurons that eventually affects secretion of FLP-1. According to our model, GRK-2 activity positively regulates chemosensory neuron activity, which in turn inhibits EGL-4 activity, modulating exploration and the arousal state of *C. elegans* through neuromodulatory signaling acting in interneurons (Fig 9B)."

Line 1031: "GRK-2 positively regulates (A) crawling and swimming by acting in premotor interneurons in a manner that depends on the D2-like dopamine receptor DOP-3 [27,28] and (B) exploration by acting in ciliated neurons in an opposite way from EGL-4. We propose a model in which GRK-2 acts in multiple ciliated sensory neurons to positively control sensory perception and (1) inhibit the activity of the EGL-4 kinase in ciliated neurons, (2) negatively regulate the neuropeptide receptor NPR-1 in RMG interneurons, and (3) negatively control the levels of FLP-1 secretion from AVK interneurons. Since the ciliated neurons and AVK interneurons do not have direct connections, we propose that GRK-2 controls FLP-1 secretion from AVK interneurons in a non-cell autonomous way through a neuromodulatory pathway. Proteins that positively regulate crawling, swimming, or exploration are shown in green and proteins that negatively regulate crawling, swimming, or exploration are shown in red. Dashed lines show indirect interactions."

Minor comments:
1. For exploration and dispersal assays, it was unclear how many trials were conducted. The numbers of animals were indicated, but were animals (and how many animals) tested on one day, multiple days (e.g., multiple trials), etc. The graphs all showed many points on the assays, but do these points represent different trials or different animals in the assays. This information should be explicitly indicated in either the Material and Methods or the figure legends (better option), and the number of trials that were performed and whether animals from different trials were combined in the graphs should be indicated.

The “Methods” section in the revised manuscript includes all the information on how many trials were conducted, what the number “n” indicates, and whether animals from different trials were combined.

Line 552: “Approximately 5-30 animals were tested per strain. Every strain was tested on two independent trials. The number “n” in figure legends indicates the total number of animals sampled (animals from the two different trials are combined).”

Line 569: “Approximately 20-40 animals were tested per strain. Every strain was tested on two independent trials. The number “n” in figure legends indicates the total number of animals sampled (animals from the different trials are combined).”

2. The specific statistical test should be indicated in each figure legend. The reader should not have to guess.

Each new figure legend in the revised manuscript includes the statistical test used for each experiment.

3. Fig. 1 legend, line 761: Panel B shows the dispersal defect in the presence, not absence, of food.

We thank the reviewer for this correction.

Line 830: “(B) *grk-2(gk268)* mutant animals have a dispersal defect in the presence of food and *dop-3(vs106)* does not rescue this phenotype.”

4. Please explain why the dispersal assay is for 1 hr, while the exploration defect is for 20 hr.

The dispersal assay measures the distance animals traveled from the middle of the plate in a short period of time. In the case of the dispersal assay we don’t want the animals to keep on wandering through the plate. This is the reason why we perform the assay for a short period of time (1h in the presence of food or 20 min in plates without food). In contrast, the exploration assay measures the ability of the animals to explore (wander) during long periods of time (overnight). We explain this in the “Methods” section:

Line 542: The exploration assay is an established assay that measures the ability of the animals to explore (wander) during long periods of time [4].”

Line 558: “The dispersal (or radial locomotion) assay is an established assay that measures the distance animals travel from the middle of the plate in a period [38]. Since the dispersal assay measures distance, it is only performed for short periods of time (1h in food or 20 min in plates without food).”

5. The data shown in graphs should also be in tables in supplementary information.

The revised manuscript includes all the data shown in graphs as Supplementary Table 3.

**Reviewer #3**: In their manuscript the authors investigate the role of G Protein-Coupled Receptor Kinase-2 (GRK-2) in exploration and quiescence behavior. They use behavioral and other assays in C. elegans in combination with genetics and rescue experiments as well as microscopy to delineate a model for how grk-2 might be working in this system to control

exploration behavior and quiescence. The authors found that GRK-2 acts in olfactory sensory neurons to promote exploration and suppress movement quiescence. From the data they conclude that GRK-2 regulatesexploration behavior in an opposite manner to NPR-1 and the neuropeptides FLP-1 and FLP-18. They study the secretion of FLP-1, a neuropeptide that regulates locomotion and found that it depends on GRK-2. Overexpression of flp-1 in turn was found to inhibit exploration behavior.

The manuscript is well written and experiments are rather well documented and the overall story makes sense. The manuscript contains a number of interesting observations and well-conducted experiments and should be interesting for many researchers interested in G protein signaling and behavior. I support publication and have very few comments regarding the manuscript, most of which are rather minor.

We thank the reviewer for the positive comments on our manuscript and for supporting publication.

1. The authors found that the ocr-2 canonical null mutant *ak47* showed an exploration defect that was not observed with the *ocr-2(ok1711)* allele which the authors presume to also be a null allele. The authors assume that one of the strains might contain a background mutation. *ok1711* is not listed in the strain list in the supplement so it is not clear which strain was used and whether this was backcrossed. Did the authors try to backcross the alleles, in an attempt to remove the background mutation? Alternatively, is there a third allele that could be used to clarify the situation? The current situation of leaving this question unresolved appears to be very dissatisfying. Also, please double check that all strains are listed in the supplement.

We agree with the reviewer's comment, and we have followed their suggestions. First, we have now included the original *ocr-2(ok1711)* strain name (VC1233) that we used in the experiment shown in new Fig 3D (Table 2). This strain is the original strain that we received from CGC without being outcrossed. Second, we outcrossed both strains VC1233 and VM396 three times and repeated the exploration assay using the outcrossed strains. Notably, we found that outcrossed strain BJH2763 *ocr-2(ak47)* is not exploration defective. This result suggests that strain VM396 contains a background mutation that makes the *ocr-2(ak47)* animals to have an exploration defect. We included these data in the revised manuscript and in new Fig S3A.

Line 234: "Surprisingly, the *ocr-2* canonical null mutant *ak47* (VM396 strain) showed an exploration defect that was not observed with the *ocr-2(ok1711)* allele (VC1233 strain). Given that *ok1711* contains a deletion of at least three exons it is presumably a null allele, suggesting that either the VC1233 *ocr-2(ok1711)* strain has a suppressor mutation that rescues the exploration defect or that the VM396 *ocr-2(ak47)* strain has an exploration defect due to a background mutation and not because of the *ocr-2(ak47)* deletion. To distinguish between these possibilities, we outcrossed strains VC1233 and VM396 and tested the exploration behavior of outcrossed strains BJH2762 *ocr-2(ok1711)* and BJH2763 *ocr-2(ak47)*. We found that strain BJH2763 *ocr-2(ak47)* did not have an exploration defect, suggesting that the *ocr-2(ak47)* mutation does not cause exploration defects and that strain VM396 has a background mutation that makes the animals exploration defective (Fig S3A)."

2. GRK-2 and EGL-4 are proposed to act in opposite ways in the ciliated neurons to regulate exploration. Could the authors explain or speculate how this might happen mechanistically?

We have modified the discussion by including a mechanistic explanation and a possible model (new Fig 9) on how GRK-2 and EGL-4 might act in opposite was to regulate exploration.

Line 434: “Given that expression of EGL-4 in the ciliated neurons reverses this suppression, we propose that both GRK-2 and EGL-4 act in ciliated neurons to control exploration but in opposite ways. We do not yet know whether the two kinases act in the same ciliated neurons to control exploration. Given that both GRK-2 and EGL-4 act in multiple ciliated neurons to regulate exploration and that other chemosensory and osmosensory mutants have similar exploration defects as *grk-2*, we support a model in which GRK-2 acts in ciliated neurons to positively control sensory perception and affect the exploration state of the animal by regulating EGL-4. We propose that in *grk-2* and cilium structure mutants, impaired sensory perception leads to increase in EGL-4 activity and decreased exploration. A similar model was supported by previous studies, which showed that EGL-4 regulates body size and locomotory states in a way depending on sensory perception [2]. Alternatively, the two kinases GRK-2 and EGL-4, might act in different ciliated neurons, leading to opposite outcomes in sensory perception and exploration.”

Line 447: “In previous work we have shown that GRK-2 regulates crawling and swimming [27,28] by possibly phosphorylating the D2-like dopamine receptor DOP-3 in premotor interneurons (Fig 9A). In the case of chemosensation and exploration, the target(s) of GRK-2, which are located in chemosensory neurons and not interneurons, are currently unknown (Fig 9B). Similarly, we do not yet know the identity of the phosphorylation target(s) for EGL-4.”

Line 462: “This result suggests that GRK-2 and EGL-4 act antagonistically to control movement quiescence outside lethargus. We propose that GRK-2 acts in ciliated neurons to control sensory perception and affect movement quiescence by negatively regulating EGL-4.”

Line 1031: “GRK-2 positively regulates (A) crawling and swimming by acting in premotor interneurons in a manner that depends on the D2-like dopamine receptor DOP-3 [27,28] and (B) exploration by acting in ciliated neurons in an opposite way from EGL-4. We propose a model in which GRK-2 acts in multiple ciliated sensory neurons to positively control sensory perception and (1) inhibit the activity of the EGL-4 kinase in ciliated neurons, (2) negatively regulate the neuropeptide receptor NPR-1 in RMG interneurons, and (3) negatively control the levels of FLP-1 secretion from AVK interneurons. Since the ciliated neurons and AVK interneurons do not have direct connections, we propose that GRK-2 controls FLP-1 secretion from AVK interneurons in a non-cell autonomous way through a neuromodulatory pathway. Proteins that positively regulate crawling, swimming, or exploration are shown in green and proteins that negatively regulate crawling, swimming, or exploration are shown in red. Dashed lines show indirect interactions.”

3. The authors state that aptf-1 mutants partially suppressed both the enhanced quiescence and reduced activity phenotypes of grk-2 mutants (Fig 5B-D). They concluded that increased sleep is partially responsible for the reduced exploration phenotype of grk-2 mutants.
Based on their data they suggested that GRK-2 acts independently of FLP-11 in regulating quiescence, and that the role of APTF-1 in adult movement quiescence is partially independent of FLP-11. “Surprisingly, we found that flp-11 mutants do not suppress the enhanced quiescence of grk-2 mutants, suggesting that APTF-1 plays a role in adult movement quiescence independent of its role in promoting FLP-11 expression. One possible explanation is that some sleep-promoting functions of RIS (or other neurons affected by aptf-1 function) do not depend on flp-11?

The authors should consider an alternative interpretation of their data: the effect of flp-11(-) is known to be weaker than that of aptf-1(-) (e.g. Turek et al eLife). Hence an alternative

explanation could be, that the assay is sensitive enough to detect a phenotype for aptf-1(-) but not for flp-11(-). This potential explanation could be especially relevant since the effect of aptf-1(-) in the assay is already rather modest. In s4c there is a trend of reduced quiescence in flp-11(-), while not scored as statistically significant, this observation supports a role for flp-11 in this process. It is usually recommended to be careful with interpreting the absence of evidence as evidence for absence.

We agree with the reviewer's comments, and we have changed the results and discussion to address this matter:

Line 299: "Alternatively, since the effect of *flp-11* on sleep is weaker than that of *aptf-1* [9], our assays might not be sensitive enough to detect a phenotype for *flp-11*."

Line 475: "One possible explanation is that APTF-1 acts through additional targets besides FLP-11, which is also supported by previous studies [9]. Alternatively, FLP-11 might be playing a smaller role than APTF-1 in adult quiescence and our assays might not be sensitive enough to detect it.

4. "Fig 5. Mutations in aptf-1 partially suppress the exploration defect, movement quiescence, and reduced activity of grk-2 mutants.
Fig 6. Mutations in npr-1 suppress the exploration defect of grk-2 mutants.
Fig 7. Mutations in flp-1 partially suppress the exploration defect of grk-2 mutants."

Repeatedly the authors write about "Mutations" when only one allele is tested. In several cases I could not find additional alleles. If only one allele is used for the experiment it would be more precise to state "mutation" as a singular.

We have followed the reviewer's suggestion and changed the word "mutations" to "mutant animals" or to "mutant" throughout the manuscript.

5. The authors might want to change their nomenclature to the more common practice of adding the p for promoter behind the gene name, ie Prab-3 to rab-3p? See also: https://wormbase.org//about/userguide/nomenclature

We have followed the reviewer's suggestion and have changed the nomenclature throughout the manuscript.
