# Supplementary material for: G protein-coupled receptor kinase-2 (GRK-2) controls exploration through neuropeptide signaling in Caenorhabditis elegans
Source: PLoS Genet. 2023 Jan 18;19(1):e1010613. doi: 10.1371/journal.pgen.1010613 (PMC9886303; doi:10.1371/journal.pgen.1010613)
Supplement: Attachment — Submitted filename: Response to Reviewers .pdf [file pgen.1010613.s010.pdf]

Point by point response: "G Protein-Coupled Receptor Kinase-2 (GRK-2) controls exploration through neuropeptide signaling in *Caenorhabditis elegans*"

Editor's comments:

Dear Dr. Topalidou,

Thank you very much for submitting your Research Article entitled 'G Protein-Coupled Receptor Kinase-2 (GRK-2) regulates exploration through neuropeptide signaling in Caenorhabditis elegans' to PLOS Genetics.

The manuscript was fully evaluated at the editorial level and by independent peer reviewers. The reviewers appreciated the attention to an important topic but identified some concerns that we ask you address in a revised manuscript.

We therefore ask you to modify the manuscript according to the review recommendations made by Reviewer #2. In particular, the concern of the relatively small "n" of many of the experiments was raised as a concern. If raising the n to closer to 30, as suggested, is not feasible in all instances, please provide a robust justification for lower sample sizes. It would also be helpful to note how many transgenic lines were assayed and pooled together in each case, as pooling data from multiple lines is generally considered more rigorous than following the behavior of just a single line that may not be fully representative of the phenotype (due to varied expression levels and mosaicism among arrays/lines).

Please also work to incorporate the suggested changes to improve the clarity of Figure 9. If some of the suggested changes are not feasible to incorporate into the figure itself, the points (e.g. speculation on the role of the additional neurons) could be added to the text of the manuscript or the figure legend. The remaining points raised by the reviewer should be able to be incorporated into the text relatively easily, and should be addressed to the extent possible.

In addition we ask that you:
1) Provide a detailed list of your responses to the review comments and a description of the changes you have made in the manuscript.
2) Upload a Striking Image with a corresponding caption to accompany your manuscript if one is available (either a new image or an existing one from within your manuscript). If this image is judged to be suitable, it may be featured on our website. Images should ideally be high resolution, eye-catching, single panel square images. For examples, please browse our archive. If your image is from someone other than yourself, please ensure that the artist has read and agreed to the terms and conditions of the Creative Commons Attribution License. Note: we cannot publish copyrighted images.

Dear Editor,

We modified the manuscript according to the recommendations made by Reviewer #2. Below we include a point-by-point response to each individual comment. As suggested by the editor, we made sure to:

1. Address the relatively small "n".

We agree with the Reviewer's general comment that "*C. elegans is relatively easy to obtain large numbers and some of the assays are not onerous to perform.*" However, some assays used in this study unfortunately require significant labor and time, making it difficult to reach a high "n". For example, the exploration assay requires one individual 6- or 10-cm plate per worm and each plate needs to be manually covered with bacteria using a glass loop. The individual worm then stays on the plate overnight and the next day the experimentalist counts the number of squares that the animal traveled through for each independent plate. Similar limitations also apply to the WorMotel assay that requires hours of recordings of individual worms.

It is worth noting that our studies reached "n" numbers that are consistent with or higher than those in prior studies. For example, Flavell et al (2020) and Raizen et al (2006) used 5-25 animals per data point in exploration assays. As in the case of the exploration assays, the "n" used in our quiescence measurements was similar to the "n" used in several previously published quiescence assays from our lab (e.g. Grubbs et al, (2020)) and other labs (e.g. Sinner et al, (2021)). Importantly, in order to minimize effects of batch-to-batch variability, we performed comparisons (and statistical testing) on worms housed on the same WorMotel device. Each WorMotel device contains 48 individually housed animals. So, with a goal of 8-12 worms per condition, we could study only 4-6 conditions per experiment. For a detailed discussion of the WorMotel approach to measure quiescence, we refer the reader to our method paper (Churgin et al, (2019)).

The relatively low "n" in previous studies indicates the robustness of the exploration and quiescence assays. The experiments described in our manuscript also show that the WorMotel and exploration assays are both robust assays that show little variability from one experiment to the other and this is apparent in the low p values that most of our experiments show. The lower "n" used in such assays suggests that the "n" used in any given assay is not necessarily fixed as it depends on the magnitude of the phenotype as well as the variance in the data. An assay that shows big effects and low variance does not need the same "n" as an assay with small effects and high variance. In other words, a lower "n" than 30 is associated with large magnitude of the phenotype and small variance in the data. Therefore, we would not expect an increase in the "n" numbers to change our results and interpretations.

We have responded to the reviewer's critique and increased sample size when possible. Specifically, we increased the number of "n" to at least 30 in all the experiments shown in Fig.1 and this change did not affect any of our conclusions drawn from the prior smaller sample size. We also increased the "n" in other experiments (Fig. 2A-F Fig. 2H-J, Fig. 3A, Fig. 4A,B, Fig. 6B,C, Fig. 6F, Fig. 7B, Fig. 7E, Fig. 8D, Fig. S1A,B, Fig. S3A,B, Fig. S4A,E), and other than small changes in statistical significance, it did not affect our conclusions. In the case of the screen (Fig. 3D) we followed the Flavell et al. (2020) protocol and used a minimum number of animals because the purpose of this experiment was to survey many sensory mutants related to *grk-2* and look for general themes, not to characterize in detail individual mutants. For this reason, we decided to screen many related mutants but use a lower "n" instead of using fewer mutants with a higher "n". The fact that many cilium structure mutants had a consistent exploration phenotype strongly supports the conclusion that a cilium structure defect leads to an exploration defect like *grk-2*.

The reviewer is correct that Supplementary Table 3 contains data that were entered sequentially representing different trials. This way we could present the data of independent repetitions altogether.

Lines 559, 575, 599: The number “n” in figure legends indicates the total number of animals sampled (animals from two or three different trials are combined).”

2. Point out how many transgenic lines were assayed and pooled together in each case.

Two independent lines were assayed in each case and the data were pooled together and presented in Sup. Table 3. In each rescue experiment the two independent lines showed similar results.

Line 543: We isolated two to three independent lines. The results shown in the figures are for two of the lines tested.

3. Improve the clarity of Figure 9 and/or of the Figure legend.

We made several changes in Figure 9 and Figure legend where we addressed the issues raised by the reviewer.

Reviewer #2: The authors examine the role of GRK-2 kinase in exploration and quiescence in C. elegans. This is a revised manuscript that addressed many of the previous concerns from the reviewers. However, in including information about how many trials were conducted and how many animals assayed in this revised version, there are several further questions that are raised. The small number of animals assayed and the small number of trials performed is somewhat surprising given that C. elegans is relatively easy to obtain large numbers and some of the assays are not onerous to perform. Generally, at least 30 animals should be assayed to accurately reflect a population, not the 5-40 shown for many strains. In the data presented in Supplementary Table 3, I assume that data were entered for the different trials sequentially (e.g., the first 10 are from trial 1 and the second 10 are from trial 2), so that the data from different trials are not bimodal?

We thank the reviewer for their suggestions for improving our manuscript. Please see our response above.

1. Fig. 2C: Why is there such variability in the off-food response? This proposed explanation should be included somewhere.

The “off-food” dispersal assays show higher variability than the exploration assays or the “on-food” dispersal assays. This is probably because the worms are more mobile in the absence of food and constantly searching for a food source, which would increase the variability of the distances they cover. To confirm the results shown in Fig. 2C we performed additional assays. Our new Fig. 2C shows that the variability remains high but the difference between the different strains is highly significant, which is the point of this experiment. We included a phrase in the manuscript explaining that the high variability is probably due to the fact that the animals are searching for food and move constantly.

Line 866: Please note the high variability in the off-food response in comparison to the on-food response (Fig. 2B). This could be because worms are more mobile in the absence of food and constantly searching for a food source, which would increase the variability of the distances they cover.

2. Fig. 2D: grk-2; osm-3;:grk-2 animals are responding as though off food, with large variability among animals. No such response is observed with grk-2 mutants alone. Why? Is the variability

due to using transgenic animals? However, these large variabilities were not seen when the same animals were assayed on food rescues.

To address whether the variability that the *grk-2; odr-3p::grk-2* animals show is due to the particular transgenic line we repeated the experiment using a different line and observed similar variability (animals 35-46 in Fig. 2D; Sup Table 3). We believe that this variability is due to the fact that these are transgenic animals (with potential mosaicism). The *grk-2; odr-3p::grk-2* animals were not used in any other experiment in the manuscript.

3. line 157: The authors should define exploration/exploring more clearly upfront. In this section the authors present data that the animals are not moving very much from the point of origin of the assay. This lack of movement could be due to decreased roaming, increased dwelling, increased quiescence, etc. The authors lump all of these process into exploration. Is this intentional? The title for this section assumes that defective exploration, which the authors define in line 251 (i.e., much later) is anything that changes movement, and then later includes quiescence into exploration (line 468). However, while their data are suggestive, the data are not sufficient yet to lump all these behaviors together.

We agree with the reviewer that the reduced exploration of *grk-2* mutant animals could be due to decreased roaming, increased dwelling, or increased quiescence. Throughout the manuscript we use the terms “exploration behavior” and “exploration assay” to relay the observation that *grk-2* mutants do not explore as much as WT. Thus, when we say that GRK-2 affects/controls exploration, we mean that it controls the exploration behavior, since *grk-2* mutants have an exploration defect, as shown using the exploration assay. We understand that this was confusing in the previous version of our manuscript. We made several changes throughout the manuscript to clarify this issue.

Line 124: This exploration behavior defect could be due to decreased roaming, increased dwelling, or increased quiescence.

4. If the same data for wild type and grk-2 mutants are shown for different figures, it should be indicated in the figures, not just in the Supplemental Tables.

The revised manuscript includes this information in the figure legends.

5. line 438: The implication is that the authors are proposing that grk-2 and egl-4 are acting in the same chemosensory neurons; this implication should be explicitly stated as in later statements, they propose an alternative hypothesis whereby the two kinases act in different neurons.

We have added a phrase explicitly proposing that GRK-2 and EGL-4 act in the same ciliated neurons to affect exploration behavior. We also deleted the alternative hypothesis since it was confusing.

Line 438: Given that expression of EGL-4 in the ciliated neurons reverses this suppression, we propose that both GRK-2 and EGL-4 act in the same ciliated neurons to control exploration but in opposite ways.

Line 1047: We propose a model in which GRK-2 acts in multiple ciliated sensory neurons to positively control sensory perception and (1) inhibit the activity of the EGL-4 kinase in the same ciliated neurons,

6. Fig. 9 is still confusing. Make the cells larger and show what genes/proteins are acting in the respective cells. For instance, GRK-2 phosphorylates DOP-3, a receptor (which should be illustrated as a transmembrane protein), in premotor interneurons to affect crawling and swimming. The authors are proposing that GRK-2, a kinase, antagonizes the action of another kinase, EGL-4, in ciliated neurons; show that in the cytoplasm as opposed to outside the cell. Among the ciliated neurons that rescue the grk-2 phenotypes, only AWB and ASH have gap junctions with RMG and only ASH also has chemical synapses with RMG. Are the authors proposing that these are the chemosensory neurons in which grk-2 is acting? That is not even indicated in the figure. How is AVK affecting the activity of motor neurons? How does grk-2 activity regulate NPR-1 activity if they are acting in different cells? Is RMG considered in the flp-1 circuit? Fig. 9 remains confusing and too simplistic.

We made several changes in Figure 9, in the figure legend, and in the text addressing the concerns of the reviewer. Our rescue experiments have shown that GRK-2 acts in multiple ciliated neurons (Fig. 2B, 2C) and not only in ASH and AWB (Figure 2E, 2F). We do not propose that GRK-2 acts only in these neurons but that it acts in other ciliated neurons as well. This is the reason why we do not indicate ASH and AWB neurons in Figure 9.

Investigating how AVKs affect the activity of motor neurons, while an interesting future direction, is out of the scope of this paper, which focuses on the role of GRK-2 in controlling exploration behavior and locomotion quiescence. Oranth et, al (2018) have suggested ways through which AVK may affect the activity of motor neurons. We pointed this out in the revised manuscript. (Line 515: AVKs were previously reported to play important roles in regulating locomotion by modulating motor neuron activity [36]; Line 1052: AVKs play critical roles in regulating locomotion by modulating motor neuron activity [36]).

Our results show that GRK-2 acts in ciliated neurons and NPR-1 in RMG neurons to control exploration, suggesting that GRK-2 regulates NPR-1 in a cell-nonautonomous way. One way that GRK-2 could regulate NPR-1 is through modulating the levels of expression of an NPR-1 neuropeptide ligand. We have emphasized those points in the revised article. (Line 489: Since GRK-2 acts in ciliated neurons and NPR-1 in RMG neurons to control exploration, this suggests that GRK-2 activity regulates NPR-1 in a cell-nonautonomous way; Line 1047: We propose a model in which GRK-2 acts in multiple ciliated sensory neurons to … negatively regulate the neuropeptide receptor NPR-1 in RMG interneurons in a cell-nonautonomous way (e.g. by regulating the level of secretion of an NPR-1 ligand)).

Neuropeptides like FLP-1 can act non-synaptically; the two neurons (e.g AVK and RMG) do not need to be in the same circuit. Also, we point out in the manuscript that NPR-1 could be activated through a different neuropeptide (e.g. FLP-18), and not through FLP-1 since *flp-1* and *flp-18* mutants do not act in an additive way to suppress the *grk-2* exploration defect.

References:

Churgin MA, Szuperak M, Davis KC, Raizen DM, Fang-Yen C, Kayser MS. Quantitative imaging of sleep behavior in *Caenorhabditis elegans* and larval Drosophila melanogaster. Nat Protoc. 2019 May;14(5):1455–88.

Flavell SW, Pokala N, Macosko EZ, Albrecht DR, Larsch J, Bargmann CI. Serotonin and the neuropeptide PDF initiate and extend opposing behavioral states in *C. elegans*. Cell. 2013 Aug 29;154(5):1023–35.

Grubbs JJ, Lopes LE, van der Linden AM, Raizen DM. A salt-induced kinase is required for the metabolic regulation of sleep. PLoS Biol. 2020 Apr;18(4):e3000220.

Oranth A, Schultheis C, Tolstenkov O, Erbguth K, Nagpal J, Hain D, et al. Food Sensation Modulates Locomotion by Dopamine and Neuropeptide Signaling in a Distributed Neuronal Network. Neuron. 2018 Dec 19;100(6):1414-1428.e10.

Raizen DM, Cullison KM, Pack AI, Sundaram MV. A Novel Gain-of-Function Mutant of the Cyclic GMP-Dependent Protein Kinase *egl-4* Affects Multiple Physiological Processes in *Caenorhabditis elegans.* Genetics. 2006 May; 173(1): 177-187.

Sinner MP, Masurat F, Ewbank JJ, Pujol N, Bringmann H. Innate Immunity Promotes Sleep through Epidermal Antimicrobial Peptides. Current Biology. 2021 Feb 8;31(3):564-577.e12.
